# Patient-Derived Head and Neck Cancer Organoids Recapitulate EGFR Expression Levels of Respective Tissues and Are Responsive to EGFR-Targeted Photodynamic Therapy

**DOI:** 10.3390/jcm8111880

**Published:** 2019-11-05

**Authors:** Else Driehuis, Sacha Spelier, Irati Beltrán Hernández, Remco de Bree, Stefan M. Willems, Hans Clevers, Sabrina Oliveira

**Affiliations:** 1 Oncode Institute, Hubrecht Institute, Royal Netherlands Academy of Arts and Sciences (KNAW) and University Medical Center Utrecht, 3584 CT Utrecht, The Netherlands; e.driehuis@hubrecht.eu (E.D.); a.spelier@students.uu.nl (S.S.); h.clevers@hubrecht.eu (H.C.); 2 Cell Biology, Department of Biology, Faculty of Science, Utrecht University, 3584 CH Utrecht, The Netherlands; 3 Pharmaceutics, Department of Pharmaceutical Sciences, Faculty of Science, Utrecht University, 3584 CG Utrecht, The Netherlands; i.beltranhernandez@uu.nl; 4 Department of Head and Neck Surgical Oncology, University Medical Center Utrecht, 3584 CX Utrecht, The Netherlands; r.debree@umcutrecht.nl; 5 Department of Pathology, University Medical Center Utrecht, 3584 CX Utrecht, The Netherlands; s.m.willems-4@umcutrecht.nl; 6 Princess Maxima Center, 3584 CS Utrecht, The Netherlands

**Keywords:** targeted photodynamic therapy, organoids, HNSCC, EGFR

## Abstract

Patients diagnosed with head and neck squamous cell carcinoma (HNSCC) are currently treated with surgery and/or radio- and chemotherapy. Despite these therapeutic interventions, 40% of patients relapse, urging the need for more effective therapies. In photodynamic therapy (PDT), a light-activated photosensitizer produces reactive oxygen species that ultimately lead to cell death. Targeted PDT, using a photosensitizer conjugated to tumor-targeting molecules, has been explored as a more selective cancer therapy. Organoids are self-organizing three-dimensional structures that can be grown from both normal and tumor patient-material and have recently shown translational potential. Here, we explore the potential of a recently described HNSCC–organoid model to evaluate Epidermal Growth Factor Receptor (EGFR)-targeted PDT, through either antibody- or nanobody-photosensitizer conjugates. We find that EGFR expression levels differ between organoids derived from different donors, and recapitulate EGFR expression levels of patient material. EGFR expression levels were found to correlate with the response to EGFR-targeted PDT. Importantly, organoids grown from surrounding normal tissues showed lower EGFR expression levels than their tumor counterparts, and were not affected by the treatment. In general, nanobody-targeted PDT was more effective than antibody-targeted PDT. Taken together, patient-derived HNSCC organoids are a useful 3D model for testing in vitro targeted PDT.

## 1. Introduction

Head and neck squamous cell carcinoma (HNSCC) is a collective term used for tumors of the stratified epithelium that lines the oral cavity, pharynx, and larynx [1]. Depending on anatomical location, tumor stage; and patient age, fitness, and comorbidities, the treatment of HNSCC can consist of surgery, radiotherapy, and chemotherapy (either alone or in combination). Early stage (stage I/II) HNSCC is usually treated with surgery or radiotherapy alone, and has a favorable prognosis. However, over 60% of patients present advanced stage disease (stage III/IV) at the time of diagnosis [2]. For these patients, treatment with curative intent consists of surgery with adjuvant radiotherapy, either alone or combined with chemotherapy, or chemoradiation with surgery in reserve for eventual salvage treatment. While surgery and radiotherapy are applied locally, these therapies may compromise important functions such as mastication and swallowing, thereby significantly decreasing the patients’ quality of life. Commonly used chemotherapeutics cisplatin and carboplatin serve as radiosensitizers, and are therefore given concurrently with radiotherapy. Although effective in a subset of patients, these treatments bring about severe side-effects [3]. Epidermal Growth Factor Receptor (EGFR) protein is overexpressed in 50%–90% of HNSCC, from which 15% carries EGFR gene amplification [4]. Accordingly, the EGFR-targeting antibody cetuximab was introduced as an alternative treatment strategy for patients ineligible for cisplatin or carboplatin treatment [4,5,6,7]. Despite these interventions, relapse rates remain around 50% for advanced stage HNSCC [8].

The limited efficacy and harsh side-effects of current treatments emphasize the need for more selective treatment strategies for HNSCC. As surgery is a main component of HNSCC treatment and tumors are often accessible, photodynamic therapy (PDT), or even the more recently explored targeted PDT, could enable such a targeted and local effect, and thereby serve as a potential therapy to treat localized HNSCC. Conventional PDT starts with the administration of a photosensitizer (PS), which is excited by locally applied light after 2–4 days. The activated PS subsequently converts oxygen to reactive oxygen species (ROS) that can damage DNA, proteins, and lipids, ultimately resulting in cell death [9,10,11]. In addition, PDT has been shown to contribute to tumor vasculature destruction and activation of the immune system [12]. Side-effects of conventional PDT (using hydrophobic PS) are common, including damage to normal surrounding tissues and skin phototoxicity. By conjugation of the PS to a tumor-targeting molecule such as an antibody, PDT can be made more tumor-specific. Although encouraging results have been obtained over the years, targeted PDT has only recently entered clinical testing [13]. The development of water-soluble PSs (such as the silicon phthalocyanine derivative IRDye700DX) has contributed to this, rendering antibody-PS conjugates more stable and eliminating the need for long spacers between antibody and PS [14]. Targeted PDT with cetuximab-IRDye700DX conjugates is currently being tested in patients diagnosed with advanced stage HNSCC (NCT02422979) [15]. The first results of this trial indicate that patients responded well to this therapy, while experiencing limited side-effects.

We have recently introduced nanobody-targeted PDT as an alternative to antibody-targeted PDT [16,17]. Nanobodies are the variable domain of a subset of antibodies that consist of heavy chains only, which are found in only a small subset of animals, including camelids [18]. Nanobodies are approximately ten times smaller than conventional antibodies, allowing for both a quicker and a more homogenous tissue penetration, as well as a faster systemic clearance when left unbound [16]. Compared to antibody-PS conjugates, the use of nanobody-PS conjugates is expected to require shorter time intervals between administration and light application (1 or 2 h, instead of day(s)), lead to more extensive tumor damage, and result in less skin photosensitivity. Using cell lines and xenografts, we have previously shown that nanobody-targeted PDT is selective for cells with high EGFR expression in vitro [16], and induces extensive tumor damage in an in vivo HNSCC model [17]. Although these results are promising, it is unclear whether EGFR expression levels of the cell lines used represent the expression of EGFR in human samples [19]. As such, it remains uncertain if findings reported thus far can be translated to the clinic.

Organoids are three-dimensional self-organizing structures that can be grown from stem cells, and recapitulate the organization, histological features, and to some extent, functional characteristics of epithelial tissues [20]. Organoids can be established with high efficiency from patient-derived material, such as surgical resections or tumor biopsies, and allow for the expansion of patient-derived tumor and normal cells in vitro. We recently developed an organoid model for HNSCC, which recapitulates morphological and genetic characteristics of this tumor type, and was found to be eligible for in vitro drug testing [21]. These 3D structures have been established from tumor and tumor-adjacent wildtype tissue, consist of proliferating squamous epithelial cells, and can be maintained in culture for over a year. Transplantation of the tumor-derived structures resulted in tumor-formation in mice, showing that these ‘mini-tumors’ retain their tumorigenic potential in vitro. Tumor-status of the organoids was confirmed using DNA sequencing (which included targeted sequencing of EGFR), and organoids were further characterized using RNA sequencing and functional drug screens. When exposed to a range of therapeutic agents, including the EGFR-targeting antibody cetuximab, we observed variable responses between organoids derived from different donors [21]. Normal organoids consisting of wildtype cells (status confirmed by DNA sequencing), on the other hand, did not result in tumor formation when transplanted.

In this study, we aim to characterize EGFR expression levels on these previously described HNSCC-derived tumor organoids [21], and compare the expression level to that of cell lines used in our previous studies. Subsequently, we investigate EGFR-targeted PDT in this 3D patient-derived model. Although, untargeted PDT has previously been tested in 3D cell cultures [22,23,24,25], to our knowledge, this is the first report of in vitro PDT in a 3D patient-derived model. Our results point to the relevance of employing patient-derived material, compared to an established cell line, for testing of targeted therapies, and support that these organoids are a useful 3D model for testing these. 

## 2. Experimental Section

### 2.1. Human Material for Organoid Cultures

The collection of patient data and tissue for the generation and distribution of organoids was performed according to the guidelines of the European Network of Research Ethics Committees (EUREC) following European and national law. The Biobank Research Ethics Committee of the UMC Utrecht (TCBio) approved the biobanking protocol: 12-093 HUB-Cancer according to the UMCU Biobanking Regulation. All donors participating in this study signed informed consent forms and can withdraw their consent at any time. Available organoids will be catalogued at www.hub4organoids.eu and can be requested at info@hub4organoids.eu.

### 2.2. Tissue Processing

Patient material was collected from pathology material in Advanced DMEM/F12 (Life Technologies, Carlsbad, CA, USA, cat. no. 12634-034), supplemented with 1× GlutaMAX (adDMEM/F12; Life Technologies, Carlsbad, CA, USA, cat. no. 12634-034), Penicillin-streptomycin (Life Technologies, Carlsbad, CA, USA, cat. no. 15140-122) and 10 mM HEPES (Life Technologies, Carlsbad, CA, USA, cat. no. 15630-056). This medium was named +/+/+. Patient material was collected in +/+/+ containing 100 µg/mL Primocin (Invivogen, San Diego, CA, USA, cat. no. ant-pm1). For normal tissue samples, excess fat or muscle tissue was removed to enrich for epithelial cells and tissue was cut into small fragments. Random pieces of approximately 5 mm^3^ were stored at −20 °C for DNA isolation. Two to four tissue pieces were fixed in formalin for histopathological analysis and immunohistochemistry, and the remainder was processed for organoid derivation. Fragments were incubated at 37 °C in 0.125% Trypsin (Sigma-Alrdrich, Saint-Louis, MO, USA, cat. no. T1426) in +/+/+ until digested. Every 10 min, the tissue suspension was sheared using 1 mL pipette. Incubation was performed for a maximum of 60 min. When complete, suspension was diluted with +/+/+ and strained over a 100 µm EasyStrainer filter (Greiner Bio-one, Alphen aan de Rijn, The Netherlands, cat. no. 542000), followed by centrifugation at 300× *g*. The resulting pellet was resuspended in ice-cold 70% 10 mg/mL cold Cultrex growth factor reduced Basement Membrane Extract (BME) type 2 (Trevigen, Gaithersburg, MD, USA, cat. no. 3533-010-02) in organoid medium, which serves as a extracellular matrix mimetic in the organoid cultures. Approximately 10,000 cells were resuspended per 40 µL of BME. Droplets of approximately 10 µL were plated on the bottom of pre-heated suspension culture plates (Greiner, Bio-one, Alphen aan de Rijn, The Netherlands, cat. no. M9312). After plating, plates were inverted and put at 37 °C for 30 min to let the BME solidify. Subsequently, prewarmed organoid medium was added to the plate. For the first week, 10 µM Rho-associated kinase (ROCK) inhibitor Y-27632 (Abmole Bioscience, Houston, TX, USA, cat. no. M1817) was added to the medium to aid outgrowth of organoids.

### 2.3. Organoid Culture

The organoid medium consisted of +/+/+, supplemented with 1× B27 supplement (Life Technologies, Carlsbad, CA, USA, cat. no. 17504-044), 1,25 mM N-acetyl-L-cysteine (Sigma-Aldrich, Saint-Louis, MO, USA, cat. no. A9165), 10 mM Nicotinamide (Sigma-Aldrich, Saint-Louis, MO, USA, cat. no. N0636), 50 ng/mL human EGF (PeproTech, London, UK, cat. no. AF-100-15), 500 nM A83-01, 10 ng/mL human FGF10 (PeproTech, London, UK, cat. no. 100-26), 5 ng/mL human FGF2 (PeproTech, London, UK, cat. no. 100-18B), 1 µM Prostaglandin E2 (Tocris Bioscience, Bristol, UK, cat. no. 2296), 3 µM CHIR 99021 (Sigma-Aldrich, Saint-Louis, MO, USA, cat no. SML1046), 1 µM Forskolin (Bio-Techne, Wiesbaden, Germany, cat. no. 1099), 4% (v/v) RSPO, and 4% (v/v) Noggin (both produced via the r-PEX protein expression platform at U-Protein Express BV, Utrecht, The Netherlands). Organoids were split between 7 and 14 days after initial plating. For passaging, organoids were collected from the plate by disrupting the BME droplets with a P1000 pipette and washed in 10 mL +/+/+. The pellet was resuspended in 1 mL of TrypLE Express (Life Technologies, Carlsbad, CA, USA, cat. no. 12605-010) for incubation at 37 °C. Digestion was closely monitored and suspension was pipetted up and down every 5 min to aid disruption of the organoids. TrypLE digestion was stopped when organoids were disrupted into single cells by adding 10 mL +/+/+. Cells were resuspended in ice-cold 70% BME in organoid medium and plated at suitable ratios (1:5 to 1:20) to allow efficient outgrowth of new organoids. Directly after splitting, 10 µM Y-27632 was added to aid outgrowth of organoids from single cells. Medium was changed every 2–3 days and organoids were split once every 1–2 weeks.

### 2.4. Cell Line Culture

Human vulvar squamous cell carcinoma A431 (CRL-1555), human cervical carcinoma cell line HeLa (CCL-2), and human embryonal kidney cell line HEK293T (CRL-3216) were purchased from the American Type Culture Collection (ATCC, LGC Standards, Germany). Human head and neck squamous cell carcinoma cell line UM-SCC-14C (14C) was kindly provided by Prof. Dr. T.E. Carey (University of Michigan, USA). All cell lines were cultured in DMEM (Life Technologies, Carlsbad, CA, USA, cat. no. 41965062) supplemented with 10% fetal calf serum, and 100 µg/mL penicillin/streptomycin (Life Technologies, Carlsbad, CA, USA, cat. no. 15140-122). Cells were cultured at 37 °C, 5% CO_2_ in a humidified atmosphere, and split with TrypLE Express for 10 min at 37 °C (Life Technologies, Carlsbad, CA, USA, cat. no. 12605-010) once a week.

### 2.5. RNA Collection

Organoids were cultured as normal. For quantification of EGFR expression, organoids were split to single cells, left to grow five days on organoid medium, and then transferred to organoid medium with physiological EGF concentration (0.63 ng/mL). Five days later, organoids were collected and washed twice with 10 mL +/+/+. RNA was extracted using RNeasy mini kit (Qiagen, Venlo, The Netherlands, cat. no. 74104) according to protocol. RNA concentrations were measured using Nanodrop.

### 2.6. cDNA Synthesis and Quantitative Polymerase Chain Reaction (PCR)

For cDNA synthesis, RNA was incubated with 50 µg/mL Oligo(dT) 15 Primer (Promega, Madison, WI, USA, cat. no. C1101) in water for 5 min at 70 °C. Subsequently GoScript Reverse Transcriptase (Promega, Madison, WI, USA, cat. no. A5003) was used according to protocol to produce cDNA. qPCR reactions were performed in 384 well format using IQ SYBR green (Bio-Rad, Hercules, CA, USA, cat. no 1708880) in the presence of 0.67 µM FW and RV primer and cDNA transcribed from 25 ng RNA. For qPCR, samples were incubated for 2 min at 95 °C and for 40 cycles at: 15 s at 98 °C, 15 s at 58 °C and 15 s at 72 °C. Results were calculated by using the ΔΔCt method.

Melt peak analysis was performed to assure that primers had no aspecific binding. The primers used are depicted in Table 1.

### 2.7. Photosensitizer Conjugation

Monovalent NB 7D12, binding domain III on EGFR, and biparatopic NB 7D12-9G8, binding domains II and III on EGFR, were produced as previously described [16]. In short, the his-tagged nanobodies were produced in E. coli BL21 and purified from the periplasmic fraction using immobilized metal affinity chromatography (IMAC) with nickel-nitrilotriacetic acid agarose. mAB cetuximab, binding domain III on EGFR, was purchased at the local hospital pharmacy. As the original ligand of EGFR, EGF also binds domain III; 7D12, 7D12-9G8, and cetuximab all compete with EGF [16]. IRDye^®^ 700DX NHS Ester (Licor, Lincoln, NE, USA, cat no. P/N 929-70010) was conjugated to cetuximab, 7D12, and 7D12-9G8, as previously described [16], thus with molar ratio for conjugation of 1:4 for all conjugates. Alexa Fluor™ 647 NHS Ester (Thermo Fisher Scientific, Waltham, MA, USA, cat. no. A20006) was similarly conjugated to cetuximab. All conjugates were checked by gel electrophoresis after conjugation to determine the percentage of free dye, and to confirm the conjugates were not degraded, they were checked on gel every 3–4 weeks after conjugation. Conjugates were solely used when free dye percentage was less than 10%. Additionally, to confirm that binding affinity was not affected after conjugation, new conjugates were tested in a binding assay on A431, as previously described [16].

### 2.8. EGFR Flow Cytometry

Organoids used for flow cytometry analysis were grown in physiological EGF medium containing 0.63 ng/mL EGF. Organoids were collected 7 days after splitting and disrupted into single cells using TrypLE. Cells were washed once with 10 mL +/+/+, counted, and subsequently incubated in FACS buffer (PBS with 5% FCS and 2 mM EDTA) containing 19 nM cetuximab-Alexa647 for one hour on ice (100 μL FACS buffer for 100,000 cells). After incubation, cells were washed once and resuspended in 100 μL FACS buffer for analysis. Just before measurements, 1 μg/mL DAPI was added to allow the identification of dead cells that were excluded from the analysis. Unstained controls were taken along for each line and A431, 14C, and Hela served as positive controls. Measurements were carried out on the BD FACSCanto II (BioRad, Hercules, CA, USA) with standard filter sets, and fluorescence intensity of cetuximab-Alexa647 was measured on the 633 nm channel. Analysis was performed using FlowJo software, BD Biosciences, Franklin Lakes, NJ, USA).

### 2.9. Immunohistochemistry

Organoids were cultured for one week on complete medium, followed by one week on physiological EGF medium. Subsequently, organoids were collected, washed twice to remove BME and incubated for at least 2 h at room temperature in 4% PFA for fixation. Organoids were subsequently processed for paraffin embedding. Paraffin sections were stained with H&E and the EGFR antibody (Invitrogen, clone 31G7, dilution 1:40, pretreatment pepsin). All stainings were performed at the pathology department of the University Medical Center Utrecht (UMCU).

### 2.10. In Vitro PDT Assay on HNSCC Organoids

Organoids were disrupted into single cells using TrypLE and manual shearing using a P1000 pipette. Single cells were subsequently plated in BME and cultured on physiological EGF medium without N-acetyl-cysteine. Two days later, BME drops were mechanically disrupted by pipetting and 1 mg/mL dispase II (Sigma-Aldrich, Saint-Louis, MO, USA, cat. No. D4693) was added to the medium to disrupt BME. Culture plates were incubated at 37 °C for 40 min to digest the BME. Subsequently, organoids were collected, washed with cold +/+/+, and filtered using a 70 μm nylon cell strainer. Organoids were counted and resuspended in 5% BME/phyiological EGF medium without N-acetyl-cysteine. Five hundred organoids were plated in a volume of 40 μL in 384 well format using the multi-drop Combi Reagent Dispenser (Thermo Fisher Scientific, Waltham, MA, USA, cat. no. 584030). Average size at the start of treatment was approximately 50 μm. PS conjugates were added using HP Tecan D300e Digital Dispenser. Conjugates were dissolved in PBS containing 0.3% Tween-20. Experiment was performed in technical triplicate. Two hours after the conjugation, 690 nm light was applied (Modulight, Tampere, Finland), using a light fluence rate of 5 mW/cm^2^ (measured by an Orion Laser power monitor, Darmstadt, Germany, cat. No. 1Z01803). A total light dose of 26 J/cm^2^ was given. Twenty-four hours after illumination, cell viability was measured using CellTiter-Glo 3D Reagent (Promega, Madison, WI, USA, cat. No. G9681) according to protocol. Luminescence readout was carried out using a Spark multimode microplate reader (Tecan, Mannedorf, Germany). Viability of wells that only received PBS/Tween for normalization was set at 100%. Viability of organoids exposed to 10 μM staurosporin was set at 0%. A separate dark control plate was made, in which for each line the highest concentration of the conjugate was added in triplicate. GraphPad Prism (GraphPad Software Inc., San Diego, CA, USA, version 8.2.0) was used to create kill curves and lines were fitted by means of the nonlinear fit option ‘log (inhibitor) vs. response -variable slope’. Concentration of the PS-conjugate was corrected for the DOC of the conjugate in order to plot on the y-axis the total amount of PS, the actual drug.

### 2.11. EGFR Overexpression Construct and Lentivirus Production

cDNA was obtained from A431 cells. EGFR open reading frame was amplified using PCR (FW primer ′5→′3: GCTAGCGCCACCATGGACTACAAGGATGACGATGACAAGATGCGACCCTCCGGGACGGC, Reverse primer ′5→′3: CACGCGTTGCTCCAATAAATTCACTGCTTT. PCR product was purified using gel extraction. Restriction digest using Nhe1 and Mlu1 allowed ligation of the EGFR open reading frame into the Addgene plasmid #50661. Production of lentivirus was performed in HEK293T cells, which were transduced with packaging plasmids and the created EGFR construct. Transduction was performed using a mixture of 300 µL PEI, 25 μg EGFR construct, and 5 mL Optimem (Thermo-Fisher, cat. Nr. 11058021) that was added to the 15 mL of DMEM/10% serum already placed on the HEK293T cells. After 8 h, medium was refreshed. Three days later, supernatant was collected and filtered using a 0.40 µm pore filter. Virus was collected by ultracentrifugation (20.000× *g*, 2 h, 4 °C). Virus derived from one 15 cm dish of HEK293T cells was resuspended in 500 μL organoid medium and stored at −80 °C until use.

### 2.12. Organoid Infection and Doxycycline-Mediated Induction of EGFR Expression

The organoids were disrupted into single cells using TrypLE, washed, and incubated with 100 μL virus suspension. Virus/cell mixture was incubated for 6 h in the presence of 1 μg/mL polybyrene. After incubation, the organoids were washed with 10 mL +/+/+ and plated in BME as usual. Three days later, the organoid medium supplemented with 1 μg/mL puromycin (InvivoGen, San Diego, CA, USA, cat. Nr. 58-58-2) was placed on the organoids. The organoids were kept on puromycin containing medium for two weeks, after which successful infection was validated using doxycycline induction. PDT was performed as previously described, except for the addition of 3 μg/mL doxycycline after splitting of the organoids two days prior to PDT. Doxycycline was also added during the PDT assay. For FACS analysis, organoids were cultured for one week in the presence of doxycycline.

## 3. Results

### 3.1. EGFR Expression Differs between Patient-Derived Organoids from Different Donors and Recapitulates EGFR Levels of Respective Tissues

Previously characterized HNSCC-derived organoids were cultured as described in [21]. Tumor-derived organoids were named T (tumor), while organoids obtained from normal tissues were named N (normal), followed by a number, which was identical to previously used in [21]. Tumor-status of the organoids was previously confirmed by DNA sequencing. This included targeted sequencing of EGFR for assessing amplification and mutations in exons 3, 7, 15, 18 to 21 (for more details on sequencing, see Supplementary Table S1). When available (i.e., in 3 out of 7 cases) organoids were established from tumor-adjacent wildtype epithelium. Wildtype status of these normal epithelium-derived organoids was confirmed by whole exome sequencing (*n* = 2) or Nutlin-3 selection (*n* = 1). Nutlin-3 is an Mdm2-agonist preventing the growth of TP53 wildtype cells [26]. Hence, Nutlin-3 sensitive lines are *TP53* wildtype. Relevant information on the organoids used in this study can be found in Table 2.

**Figure 1:**
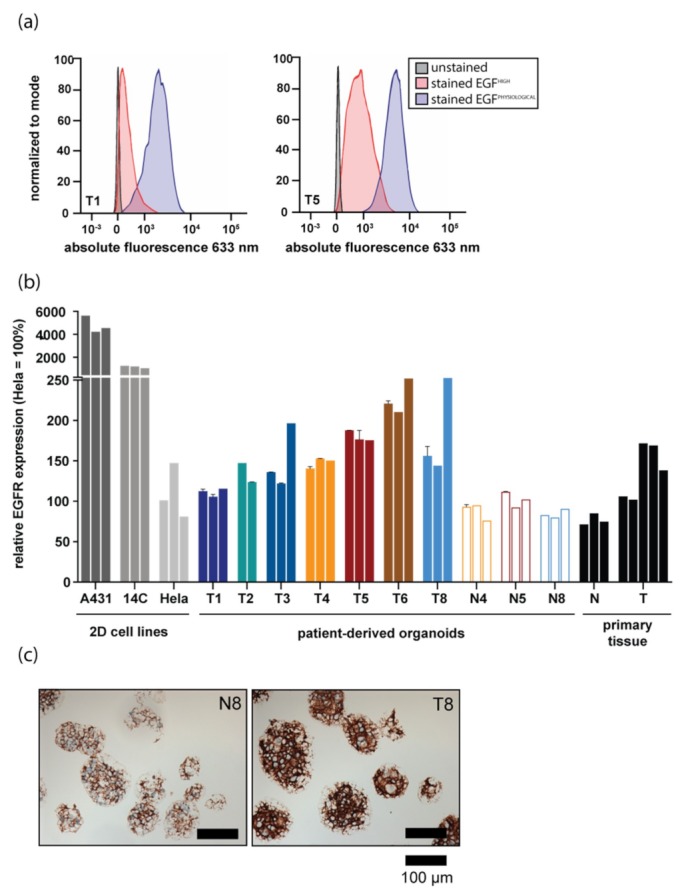
Epidermal Growth Factor Receptor (EGFR) expression differs between patient-derived organoids from different donors and recapitulates EGFR levels of respective tissues. (**a**) EGFR protein expression detected by flow cytometry. EGFR expression of organoids grown in either physiological Epidermal Growth Factor (EGF) (blue peak) or high EGF (ref peak) medium is shown for two HNSCC organoid lines. An unstained control is shown in black. (**b**) EGFR protein expression measured by flow cytometry in 2D cell lines commonly used in in vitro EGFR-targeted PDT studies, organoid lines derived from HNSCC patients both normal and tumor, and in primary tissue samples. For organoids, the experiment was performed in technical duplicate (error bars) and biological triplicate (individual bars). EGFR expression was stable over time, as biological replicates were measured two months apart. EGFR protein levels are shown relative to HeLa cells (set at 100%). Results of tumor organoids are shown in filled bars, results of wildtype organoids are shown in clear, outlined bars. For primary tissues, each bar represents EGFR expression on a tissue sample derived from an individual patient. (**c**) EGFR immunohistochemical staining performed on N8 and T8 organoids. Scalebar, 100 µm.

To assess EGFR expression levels in HNSCC organoids, both quantitative PCR and flow cytometry were performed. EGFR messenger RNA was detectable in all tested organoid lines, although expression varied between organoids derived from different donors (Figure S1A). Interestingly, lowering the level of EGF in organoid culture medium to the levels detected in human serum (hereafter called ‘physiological EGF’), resulted in upregulation of EGFR expression in all, but two lines (Figure S1B). This finding was in line with the fact that EGFR protein on organoids could initially not be detected by flow cytometry, whereas it was detectable on control cell lines overexpressing EGFR. Indeed, EGFR protein levels were increased upon culture of organoids in physiological EGF medium (Figure 1a). In line with variable EGFR expression in primary tumors [27], EGFR protein levels varied between organoids derived from different donors (Figure 1b). EGFR protein levels detected on HNSCC-derived organoids were lower than those observed on cell lines expressing high (A431) and moderate (14C) EGFR levels (indicated by ‘2D cell lines’ in Figure 1b). Expression is shown relative to HeLa cells, which have been reported to have physiological levels of EGFR expression. All these cell lines have previously been used to assess efficacy and selectivity of EGFR-targeted PDT in vitro [16,17].

As organoid culture allows for the expansion of corresponding normal tissue in culture, EGFR levels of organoid grown from surrounding normal tissues were also measured. For three donors, EGFR levels on both normal and tumor organoids were compared. In all cases, EGFR protein levels detected on tumor cells were higher than those detected on matched normal organoids (Figure 1b). Importantly, EGFR expression levels in organoids (colored bars) were comparable to EGFR levels of primary patient material samples (black bars indicated with ‘primary tissue’, Figure 1b). The observed differences in EGFR expression between normal and tumor organoids were confirmed using immunohistochemistry (Figure 1c).

### 3.2. Organoid Response to EGFR-Targeted PDT Is Donor-Dependent and Tumor-Specific

**Figure 2:**
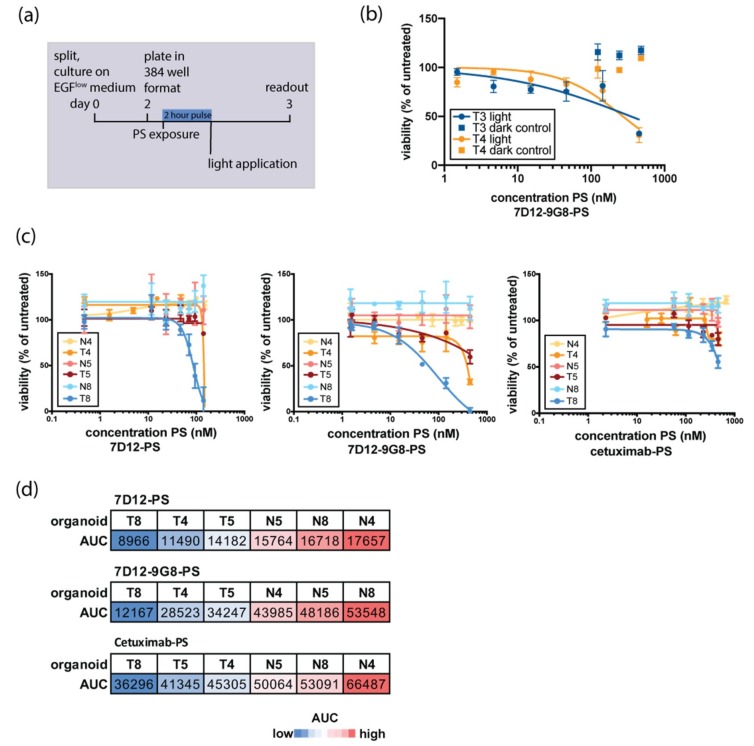
Organoid response to in vitro EGFR-targeted PDT is donor-dependent and tumor-specific. (**a**) Schematic outline of the experimental set-up of organoid in vitro PDT. Organoids are disrupted into single cells, recovered for two days on medium containing physiological EGF, and subsequently filtered, counted, and plated into a 384 well format. A two-hour exposure to EGFR-targeting nanobody-PS or antibody-PS conjugates was followed by a light-dose activating the PS. Twenty-four hours later, cell viability was assessed. (**b**) EGFR-targeting conjugates used in this study did not show toxicity without activation of the PS. Here, toxicity of 7D12-9G8 in T3 and T4 is shown as an example. (**c**) Response to EGFR-targeted PDT of matched normal and tumor organoid pairs. Response to 7D12-PS, 7D12-9G8-PS, and cetuximab-PS is shown for N4 and T4 (orange), N5 and T5 (red), and N8 and T8 (blue). Normal organoids are depicted in a lighter shade of the same color than their tumor counterparts. (**d**) Quantification of organoid response to EGFR-targeted PDT shown in Figure 2c Area under the curve (AUC) is used as a readout for response to EGFR-targeted PDT. Blue indicates low AUC-values (response to therapy), whereas red indicates high AUC values (no response to therapy).

Existing PDT protocols of 2D cell lines were adjusted to make them suitable for organoid screens (Figure 2a). All experiments were performed with cetuximab-PS (which is currently tested in the clinic), and the nanobody-PS conjugates 7D12-PS and 7D12-9G8-PS, that were used in our previous studies [16]. 7D12 is a monovalent EGF-competing nanobody, whereas 7D12-9G8-PS is a biparatopic EGF-competing nanobody, consisting of two genetically fused monomeric nanobodies targeting two different epitopes on EGFR. In vitro, biparatopic nanobodies have been shown to be more potent than monovalent nanobodies [16], as they can carry more PS per nanobody and can promote receptor clustering, which leads to faster EGFR endocytosis and thus, conjugate internalization [28].

Upon exposure to EGFR-targeted PDT, organoids were killed by concentrations of PS-conjugate that did not result in cell death without PS-activating light exposure (Figure 2b, shown for 7D12-9G8-PS, but found for all used conjugates). As expression levels are lower in patient-derived organoids than in the cell lines where efficacy of these nanobody-PS conjugates has been observed before [16], these findings are encouraging and of clinical relevance.

Tumor and wildtype organoids established from the same patient were subjected in parallel to EGFR-targeted PDT. In all pairs tested, tumor organoids were found to be more sensitive to PDT than their wildtype counterparts (Figure 2c). Using area under the curve (AUC) as a quantitative measure of in vitro killing, tumor organoids clustered separately from wildtype organoids, for all PS-conjugates tested (Figure 2d). Although numbers are limited, these findings are encouraging and provide the first indication that cancer cells derived from patient material can be selectively killed by targeted PDT, leaving normal cells unaffected. For all organoids tested, the effect of nanobody-targeted PDT was more pronounced than that of antibody-targeted PDT. A comparison between the two nanobody-PS conjugates points to the treatment with the biparatopic nanobody 7D12-9G8-PS, as this is the most effective in most of the organoids tested.

### 3.3. Organoid Response to EGFR-Targeted PDT Correlates with EGFR Expression Levels

**Figure 3:**
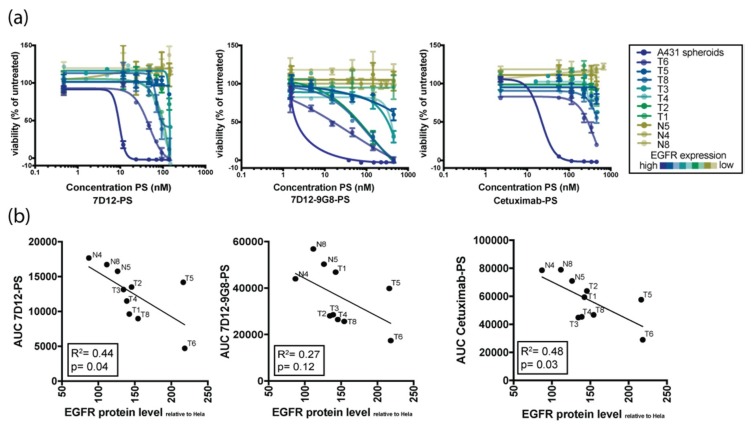
Organoid response to in vitro EGFR-targeted PDT correlates with EGFR expression levels. (**a**) Sensitivity of HNSCC organoids with variable EGFR expression to PDT using conjugates 7D12-PS (first panel), 7D12-9G8-PS (second panel), and cetuximab-PS (third panel). Color of the lines indicate the relative EGFR expression level, detected by FACS analysis (blue = highest expression, yellow = lowest expression). (**b**) Correlation plots showing the relation between EGFR expression on the *x*-axis and response to PDT therapy, as indicated by area under the curve (AUC) on the *y*-axis. The first panel shows the AUC for 7D12-PS, second panel for 7D12-9G8-PS, and the third panel for cetuximab-PS.

The number of patient-derived tumor organoids was further expanded and their response to targeted PDT assessed (Figure 3). Organoids derived from different patients showed variable responses to EGFR-targeted PDT. Comparison of EGFR protein levels and response to therapy revealed a positive correlation between these two variables, confirming results previously obtained in 2D cell lines [16]. Organoids that express higher protein levels of EGFR were more sensitive to EGFR-targeted PDT, regardless of the PS-conjugate used (Figure 3a,b). Since organoids express EGFR at comparable levels to primary patient tissue, these results are clinically relevant, as they suggest that EGFR levels could be a predictor for EGFR-targeting PDT. The response of all individual organoids (both tumor and normal) to the three used PS-conjugates is shown in Figure S2.

### 3.4. Induction of EGFR Expression Increases Sensitivity to EGFR-Targeted PDT

**Figure 4:**
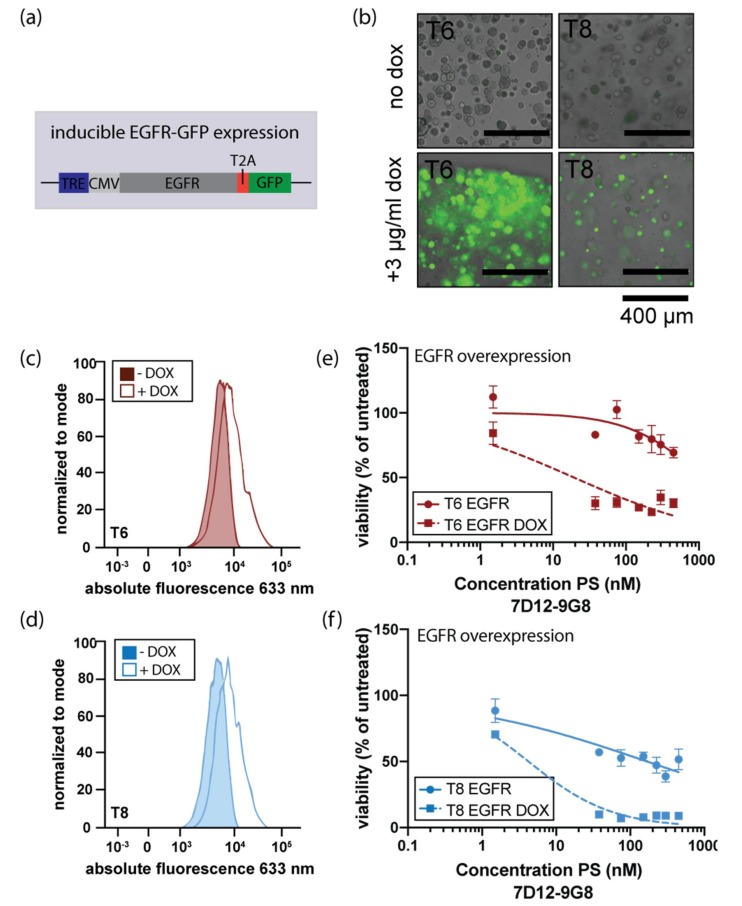
Induced EGFR expression enhances the response to EGFR-targeted PDT. (**a**) Schematic outline of lentiviral infector used to create organoid lines that can be induced to overexpress EGFR (TRE: tetracycline responsive element, CMV: cytomegalovirus promotor). The TRE and CMV promotor precede the open reading frame encoding EGFR and GFP, separated by a T2A sequencing, assuring generation of separate mRNA molecules for EGFR and GFP. (**b**) Merged brightfield/immunofluorescence images of organoids after two-day administration of doxycycline. Scalebar 400 μm. (**c**) and (**d**). Effect of doxycycline-mediated EGFR overexpression on EGFR protein levels in organoid lines T6 and T8, respectively. Colored peaks indicate uninduced expression, lined peaks indicate induction of EGFR expression. (**e**) and (**f**). EGFR-targeted PDT using nanobody 7D12-9G8-PS in EGFR overexpressing organoid lines T6 and T8. Organoids were either doxycycline-induced (squared symbols, dashed line) or uninduced (round symbols, solid line) and exposed to PDT as previously described.

Based on the observed correlation between EGFR expression and the targeted PDT response, we set out to verify whether an increase in EGFR expression would result in increased sensitivity to EGFR-targeted PDT. For this, a doxycycline-inducible EGFR expression construct was introduced into two HNSCC organoids, T6 and T8 (Figure 4a). Subsequent doxycycline-induction resulted in GFP expression (A GFP-coding sequence was cloned after EGFR in the used construct), indicating that the induction was effective (Figure 4b). After doxycycline treatment, EGFR protein levels increased in both lines (Figure 4c). Lastly, EGFR overexpression resulted in increased sensitivity to targeted PDT (Figure 4d). This data confirms that EGFR-targeted PDT is most effective in organoids having high expression of EGFR, showing a causal relation between EGFR levels and sensitivity to EGFR-targeted PDT.

## 4. Discussion

The first aim of this study was to characterize EGFR expression in HNSCC patient-derived organoids and compare the expression level to that of cell lines used in our previous studies. EGFR levels in organoids were found to recapitulate EGFR expression levels of both tumor and normal patient material samples, even when retained in culture. Notably, EGFR expression levels were found to be lower in patient-derived organoids than in cell lines where efficacy of these nanobody-PS conjugates has been observed before [16]. The EGFR levels detected on organoids were comparable to expression levels detected on HeLa cells, a cell line that has been found to be unaffected by nanobody-targeted PDT under specific conditions [15]. Subsequently, we aimed to investigate EGFR-targeted PDT in this novel 3D model; in the lower levels of EGFR expression, EGFR-targeted PDT was shown to be effective in these patient-derived organoids. Tumor organoids were more sensitive to PDT than their corresponding wildtype counterparts, suggesting that, with appropriate dosing, this therapy is expected to leave the normal epithelium surrounding the tumor unaffected. EGFR levels between organoids from different patients varied, and could be correlated with response to EGFR-targeted PDT. Higher levels of EGFR protein correlated with a better response to this therapy in vitro. Finally, we aimed to verify the correlation between EGFR expression levels and PDT efficacy. For that, inducible EGFR overexpression was employed, which resulted in increased PDT efficacy.

The known clinical need for effective and selective local treatment of HNSCC, and the complexity of conventional PDT protocols used in the clinic encourages the development of more targeted PDT approaches. This study complements others that have shown selective and extensive tumor damage as a result of EGFR-targeted PDT [29,30,31,32,33,34]. However, here we have taken the unique approach of testing PDT in (matching) patient-derived tumor and normal cells. To our knowledge, this is the first report of in vitro PDT in a 3D patient-derived model, although several studies have investigated PDT or targeted PDT in spheroid models grown from 2D cell lines [22,23,24,25]. When compared to the 2D cultures of which they were derived, these 3D spheroids certainly better recapitulate a mass of tumor cells in vivo. However, this study highlights the possible differences in target expression levels between such 3D spheroids and patient-derived organoids, where the expression detected in the latter was found to better recapitulate expression levels detected on primary tissue. Thus, the use of patient-derived models should be encouraged in the development and evaluation of targeted therapies.

Gene amplification of EGFR is reported in 15% of HNSCC patients [4], although EGFR protein overexpression has been reported in up to 90% of tumors [5]. Here, tumor cells showed increased EGFR expression levels compared to their wildtype counterparts, despite the fact that none of the tested organoids showed any evidence for EGFR amplification [21]. These findings are in line with previously mentioned statistics and support the hypothesis that EGFR protein overexpression is not only driven by gene amplification. It would be of interest to test EGFR-targeted PDT in organoids derived from a tumor carrying EGFR amplification, to see if responses are comparable to those observed in the organoids reported here.

In this work, we confirmed the correlation that was previously observed between EGFR expression and response to EGFR-targeting PDT [16,17]. Here, we confirm this correlation in patient-derived organoids. These findings suggest that high EGFR expression in tumor tissue might be used as a predictive marker for response to EGFR-targeted PDT. The potential of EGFR expression as a biomarker for other EGFR-targeted therapies was previously investigated [5]. Such studies found that EGFR expression levels could not predict response to the EGFR-blocking antibody cetuximab. This is likely explained by commonly found downstream mutations in the EGFR pathway, which will negate the effect of cetuximab competing with EGF for EGFR binding, even in EGFR overexpressing tumors [4]. Since the effect of EGFR-targeted PDT is independent from EGFR downstream signaling and mutations, EGFR expression could potentially be a suitable biomarker for response to EGFR-targeted PDT. EGFR-targeted PDT is expected to be an effective treatment option, even for tumors carrying EGFR-downstream mutations.

Cetuximab-targeted PDT is currently tested in clinical trials (NCT02422979). Initial findings are encouraging and show responses to therapy, with limited side-effects [15]. Here, no inclusion criteria were applied based on EGFR expression levels. It would be of value to see to what extent EGFR levels can explain differential responses to therapy in this patient cohort. To determine if organoids can aid personalized decision-making, it would be interesting to establish organoids from patients receiving EGFR-targeted PDT. As such, it would be possible to compare in vitro organoid responses to corresponding patient responses and see if organoids hold predictive potential in EGFR-targeted PDT.

Even though PDT is a local therapy, evidences have pointed to the potential of it triggering an immune response that can lead to systemic effects and even a memory to protect patients from tumor recurrences [35]. For patients already diagnosed with metastatic disease, a combination of the standard systemic chemotherapy with PDT could be foreseen. Such combinations of chemotherapy and PDT have already been explored in several preclinical and clinical studies (as reviewed in [36]). Overall, the addition of PDT has resulted in increased response rates when compared to chemotherapy alone, even in advanced disease where metastasis are present [36]. Combination treatment often results in more severe side-effects than single agent therapy, thereby hampering clinical applicability. As the use of targeted PDT has shown less side-effects than conventional PDT [15], it is suggested that the introduction of targeted PDT in combination with chemotherapy will be better tolerated than conventional PDT combined with chemotherapy.

The efficiency of organoid culture allows for the establishment of matching organoid pairs, where corresponding normal oral mucosa tissue can be grown in parallel to the adjacent tumor tissue. Here, we tested three matching organoid pairs, and found in all cases that wildtype organoids showed no response to EGFR-targeted PDT. This is likely due to the lower EGFR expression levels detected on wildtype cells, although others have shown that tumor cells also produce more ROS than wildtype cells, which might also influence PDT responses [37,38].

Patient-derived organoids create the opportunity to compare the response of both tumor and matching wildtype cells. These allow to study the specificity of EGFR-targeted PDT better than, for example, transplantation of patient-derived tumor cells in mice, where the species difference can contribute to differential response of (human) tumor and (mouse) wildtype cells. On the contrary, in vivo studies are better suited than in vitro models to evaluate distribution and the effect of bio-availability of PS-conjugates. A possible solution could be transplantation of both the normal and tumor organoid pairs in order to investigate these aspects in vivo [21].

In this study, we used two different nanobodies: monovalent 7D12 and biparatopic 7D12-9G8. In vitro, biparatopic nanobodies have been shown to be more potent than monovalent nanobodies [16], as they can carry more PS per nanobody, and their binding to EGFR results in receptor clustering, leading to faster EGFR (and conjugate) endocytosis [28]. The internalization of nanobody-PS and also of antibody-PS has been correlated with increased cellular damage [39,40]. However, in vivo, only small differences have been observed thus far between these two nanobody formats, where 7D12-PS has shown more reproducible tumor damage than 7D12-9G8-PS. As 7D12 is smaller than 7D12-9G8, tissue penetration of this nanobody is suggested to be more rapid and more homogenous [41]. Compared to treatment with antibody-PS, nanobody–PS results in less variation in the extent of damage induced and in an increase in tumor damage [17]. These results have been correlated to the larger size of the antibody, hampering a homogenous distribution in vivo. Also, in organoids, cetuximab–PS was found to be less effective than any of the two nanobodies (Figure S2). In contrast to previously described in vivo studies, we found that, in vitro, the biparatopic nanobody more effectively killed HNSCC cells than the monovalent nanobody. This is likely due to the small dimensions of the organoids employed, and the fact that this set up does not reflect systemic distribution. Nevertheless, besides our study focused on targeted PDT, organoids are gaining increasingly more attention as realistic representations of tumors that enable effective drug screens, with a variety of drugs [21,42,43,44,45,46,47,48,49,50,51,52].

While the predictive potential of patient-derived organoids has been shown earlier for cystic fibrosis [53,54], recent data shows that organoids also hold predictive potential in oncological therapeutic context [21,44,45,46,48]. In these studies, a correlation between patient response and the response of matching organoids that were exposed to the same therapy was observed. Although numbers are limited, future studies will show if such a correlation holds true in a larger cohort of patients and for more tumor types.

## 5. Conclusions

Here we present HNSCC-derived organoids as a clinically relevant 3D model for in vitro targeted PDT. We show that EGFR expression levels detected on patient-derived HNSCC organoids closely recapitulate EGFR expression detected in primary tissue. EGFR expression levels were found to correlate to the response to EGFR-targeted PDT. Wildtype organoids grown from tumor-adjacent normal tissues showed lower EGFR expression levels than their tumor counterparts, and were not affected by PDT. Taken together, patient-derived organoids are shown to be a useful model for EGFR-targeted PDT research.

## Figures and Tables

**Table 1:** Primer sequences used for quantitative Polymerase Chain Reaction.

Primer	Sequence ′5→′3
Human EGFR FW	AGGCAGGAGTAACAAGCTCAC
Human EGFR RV	ATGAGGACATAACCAGCCACC
Human GAPDH FW	GGAGCGAGATCCCTCCAAAAT
Human GAPDH RV	GGCTGTTGTCATACTTCTCATCG

EGFR: Epidermal Growth Factor Receptor, FW: Forward, RV: Reverse, GAPDH: Glyceraldehyde 3-phosphate dehydrogenase.

**Table 2:** Patient information corresponding to the organoid lines used in this study. From left to right, columns indicate: patient gender, patient age at diagnosis, tumor location, pretreatment, Human Pappiloma Virus (HPV) status of tumor, type of DNA sequencing used to confirm tumor status of the organoid line, and availability of organoids from only tumor (T) or both normal epithelium and tumor (N/T).

Organoid	Gender	Age	Tumor Location	HPV Status	Sequencing to Confirm Tumor Status	Tumor Status Confirmed?	N/T
1	male	61	tongue	negative	oncopanel	Yes	T
2	female	90	larynx	negative	oncopanel	Yes	T
3	female	83	larynx	negative	oncopanel	Yes	T
4	male	60	tongue	negative	oncopanel	Yes	N/T
5	male	80	parotid gland	negative	exome sequencing	Yes	N/T
6	male	82	oral cavity	negative	oncopanel	Yes	T
8	female	70	gingiva	negative	exome sequencing	Yes	N/T

## Supplementary Materials

The following are available online at https://www.mdpi.com/2077-0383/8/11/1880/s1, Figure S1: EGFR mRNA expression differs between patient-derived organoid lines and is increased upon medium change to physiological EGF levels, Figure S2: Comparison of different PS carriers in EGFR-targeted PDT.

## References

[1] Squier C., Kremer M. (2001). Biology of oral mucosa and esophagus. J. Natl. Cancer Inst. Monogr..

[2] Tijink B.M., Buter J., de Bree R., Giaccone G., Lang M.S., Staab A., Leemans C.R., van Dongen G.A.M.S. (2006). A phase I dose escalation study with anti-CD44v6 bivatuzumab mertansine in patients with incurable squamous cell carcinoma of the head and neck or esophagus. Clin. Cancer Res..

[3] Guan J., Li Q., Zhang Y., Xiao N., Chen M., Zhang Y., Li L., Chen L. (2016). A meta-analysis comparing cisplatin-based to carboplatin-based chemotherapy in moderate to advanced squamous cell carcinoma of head and neck (SCCHN). Oncotarget.

[4] The Cancer Genome Atlas Network (2015). Comprehensive genomic characterization of head and neck squamous cell carcinomas. Nature.

[5] Bossi P., Resteghini C., Paielli N., Licitra L., Pilotti S., Perrone F. (2016). Prognostic and predictive value of EGFR in head and neck squamous cell carcinoma. Oncotarget.

[6] Mandic R., Rodgarkia-Dara C.J., Zhu L., Folz B.J., Bette M., Weihe E., Neubauer A., Werner J.A. (2006). Treatment of HNSCC cell lines with the EGFR-specific inhibitor cetuximab (Erbitux^®^) results in paradox phosphorylation of tyrosine 1173 in the receptor. FEBS Lett..

[7] Bonner J.A., Harari P.M., Giralt J., Azarnia N., Shin D.M., Cohen R.B., Jones C.U., Sur R., Raben D., Jassem J. (2006). Radiotherapy plus cetuximab for squamous-cell carcinoma of the head and neck. N. Engl. J. Med..

[8] Argiris A., Karamouzis M.V., Raben D., Ferris R.L. (2008). Head and neck cancer. Lancet (London, England).

[9] Plaetzer K., Krammer B., Berlanda J., Berr F., Kiesslich T. (2009). Photophysics and photochemistry of photodynamic therapy: Fundamental aspects. Lasers Med. Sci..

[10] Trachootham D., Alexandre J., Huang P. (2009). Targeting cancer cells by ROS-mediated mechanisms: A radical therapeutic approach?. Nat. Rev. Drug Discov..

[11] Curtin J.F., Donovan M., Cotter T.G. (2002). Regulation and measurement of oxidative stress in apoptosis. J. Immunol. Methods.

[12] Agostinis P., Berg K., Cengel K.A., Foster T.H., Girotti A.W., Gollnick S.O., Hahn S.M., Hamblin M.R., Juzeniene A., Kessel D. (2011). Photodynamic therapy of cancer: An update. CA Cancer J. Clin..

[13] Mew D., Wat C.-K., Towers G.H., Levy J.G. (1983). Photoimmunotherapy: Treatment of animal tumors with tumor-specific monoclonal antibody-hematoporphyrin conjugates. J. Immunol..

[14] Van Dongen G.A., Visser G.W., Vrouenraets M.B. (2004). Photosensitizer-antibody conjugates for detection and therapy of cancer. Adv. Drug Deliv. Rev..

[15] Cognetti D., Curry J.M., Gillenwater A.M., William W.N., Kochuparambil S.T., McDonald D., Fidler M., Stenson K.M., Vasan N.R., Razaq M.A. (2018). A phase 2a, multicenter, open-label study of RM-1929 photoimmunotherapy in patients with recurrent head and neck cancer. Int. J. Radiat. Oncol..

[16] Heukers R., van Bergen en Henegouwen P.M., Oliveira S. (2014). Nanobody-photosensitizer conjugates for targeted photodynamic therapy. Nanomedicine.

[17] Van Driel P.B.A.A., Boonstra M.C., Slooter M.D., Heukers R., Stammes M.A., Snoeks T.J.A., De Bruijn H.S., Van Diest P.J., Vahrmeijer A.L., van Bergen En Henegouwen P.M.P. (2016). EGFR targeted nanobody-photosensitizer conjugates for photodynamic therapy in a pre-clinical model of head and neck cancer. J. Control. Release.

[18] Hamers-Casterman C., Atarhouch T., Muyldermans S., Robinson G., Hammers C., Songa E.B., Bendahman N., Hammers R. (1993). Naturally occurring antibodies devoid of light chains. Nature.

[19] Carpenter G., Cohen S. (1979). Epidermal growth factor. Annu. Rev. Biochem..

[20] Kretzschmar K., Clevers H. (2016). Organoids: Modeling Development and the Stem Cell Niche in a Dish. Dev. Cell.

[21] Driehuis E., Kolders S., Spelier S., Lõhmussaar K., Willems S.M., Devriese L.A., de Bree R., de Ruiter E.J., Korving J., Begthel H. (2019). Oral mucosal organoids as a potential platform for personalized cancer therapy. Cancer Discov..

[22] Broekgaarden M., Rizvi I., Bulin A.L., Petrovic L., Goldschmidt R., Massodi I., Celli J.P., Hasan T. (2018). Neoadjuvant photodynamic therapy augments immediate and prolonged oxaliplatin efficacy in metastatic pancreatic cancer organoids. Oncotarget.

[23] Zuchowska A., Jastrzebska E., Chudy M., Dybko A., Brzozka Z. (2017). 3D lung spheroid cultures for evaluation of photodynamic therapy (PDT) procedures in microfluidic lab-on-a-chip system. Anal. Chim. Acta.

[24] Sato K., Hanaoka H., Watanabe R., Nakajima T., Choyke P.L., Kobayashi H. (2015). Near infrared photoimmunotherapy in the treatment of disseminated peritoneal ovarian cancer. Mol. Cancer Ther..

[25] Millard M., Yakavets I., Piffoux M., Brun A., Gazeau F., Guigner J.-M., Jasniewski J., Lassalle H.-P., Wilhelm C., Bezdetnaya L. (2018). mTHPC-loaded extracellular vesicles outperform liposomal and free mTHPC formulations by an increased stability, drug delivery efficiency and cytotoxic effect in tridimensional model of tumors. Drug Deliv..

[26] Vassilev L.T., Vu B.T., Graves B., Carvajal D., Podlaski F., Filipovic Z., Kong N., Kammlott U., Lukacs C., Klein C. (2004). In vivo activation of the p53 pathway by small-molecule antagonists of MDM2. Science.

[27] Kalyankrishna S., Grandis J.R. (2006). Epidermal growth factor receptor biology in head and neck cancer. J Clin. Oncol..

[28] Heukers R., Vermeulen J.F., Fereidouni F., Bader A.N., Voortman J., Roovers R.C., Gerritsen H.C., Van Bergen En Henegouwen P.M.P. (2013). Endocytosis of EGFR requires its kinase activity and N-terminal transmembrane dimerization motif. J. Cell Sci..

[29] Gijsens A., Missiaen L., Merlevede W., De Witte P. (2000). Epidermal growth factor-mediated targeting of chlorin e6 selectively potentiates its photodynamic activity. Cancer Res..

[30] Hemming A.W., Davis N.L., Dubois B., Quenville N.F., Finley R.J. (1993). Photodynamic therapy of squamous cell carcinoma. An evaluation of a new photosensitizing agent, benzoporphyrin derivative and new photoimmunoconjugate. Surg. Oncol..

[31] Kameyama N., Matsuda S., Itano O., Ito A., Konno T., Arai T., Ishihara K., Ueda M., Kitagawa Y. (2011). Photodynamic therapy using an anti-EGF receptor antibody complexed with verteporfin nanoparticles: A proof of concept study. Cancer Biother. Radiopharm..

[32] Master A., Malamas A., Solanki R., Clausen D.M., Eiseman J.L., Sen Gupta A. (2013). A cell-targeted photodynamic nanomedicine strategy for head and neck cancers. Mol. Pharm..

[33] Springa B.Q., Abu-Yousif A.O., Palanisami A., Rizvi I., Zheng X., Mai Z., Anbil S., Sears R.B., Mensah L.B., Goldschmidt R. (2014). Selective treatment and monitoring of disseminated cancer micrometastases in vivo using dual-function, activatable immunoconjugates. Proc. Natl. Acad. Sci. USA.

[34] Mitsunaga M., Ogawa M., Kosaka N., Rosenblum L.T., Choyke P.L., Kobayashi H. (2011). Cancer cell-selective in vivo near infrared photoimmunotherapy targeting specific membrane molecules. Nat. Med..

[35] Maeding N., Verwanger T., Krammer B. (2016). Boosting Tumor-Specific Immunity Using PDT. Cancers (Basel)..

[36] Luo D., Carter K.A., Miranda D., Lovell J.F. (2017). Chemophototherapy: An emerging treatment option for solid tumors. Adv. Sci..

[37] Schumacker P.T. (2006). Reactive oxygen species in cancer cells: Live by the sword, die by the sword. Cancer Cell.

[38] Szatrowski T.P., Nathan C.F. (1991). Production of large amounts of hydrogen peroxide by human tumor cells. Cancer Res..

[39] Carcenac M., Dorvillius M., Garambois V., Glaussel F., Larroque C., Langlois R., Hynes N.E., Van Lier J.E., Pèlegrin A. (2001). Internalisation enhances photo-induced cytotoxicity of monoclonal antibody-phthalocyanine conjugates. Br. J. Cancer.

[40] Vrouenraets M.B., Visser G.W.M., Stigter M., Oppelaar H., Snow G.B., Van Dongen G.A.M.S. (2001). Targeting of aluminum (III) phthalocyanine tetrasulfonate by use of internalizing monoclonal antibodies: Improved efficacy in photodynamic therapy. Cancer Res..

[41] Beltrán Hernández I., Rompen R., Rossin R., Xenaki K.T., Katrukha E.A., Nicolay K., van Bergen en Henegouwen P., Grüll H., Oliveira S., Beltran Hernandez I. (2019). Imaging of tumor spheroids, dual-isotope SPECT, and autoradiographic analysis to assess the tumor uptake and distribution of different nanobodies. Mol. Imaging Biol..

[42] Van De Wetering M., Francies H.E., Francis J.M., Bounova G., Iorio F., Pronk A., Van Houdt W., Van Gorp J., Taylor-Weiner A., Kester L. (2015). Prospective derivation of a living organoid biobank of colorectal cancer patients. Cell.

[43] Mullenders J., de Jongh E., Brousali A., Roosen M., Blom J.P.A., Begthel H., Korving J., Jonges T., Kranenburg O., Meijer R. (2019). Mouse and human urothelial cancer organoids: A tool for bladder cancer research. Proc. Natl. Acad. Sci. USA.

[44] Ooft S.N., Weeber F., Dijkstra K.K., McLean C.M., Kaing S., van Werkhoven E., Schipper L., Hoes L., Vis D.J., van de Haar J. (2019). Patient-derived organoids can predict response to chemotherapy in metastatic colorectal cancer patients. Sci. Transl. Med..

[45] Sachs N., de Ligt J., Kopper O., Gogola E., Bounova G., Weeber F., Balgobind A.V., Wind K., Gracanin A., Begthel H. (2018). A living biobank of breast cancer organoids captures disease heterogeneity. Cell.

[46] Tiriac H., Belleau P., Engle D.D., Plenker D., Deschênes A., Somerville T., Froeling F.E.M., Burkhart R.A., Denroche R.E., Jang G.-H. (2018). Organoid profiling identifies common responders to chemotherapy in pancreatic cancer. Cancer Discov..

[47] Kopper O., de Witte C.J., Lohmussaar K., Valle-Inclan J.E., Hami N., Kester L., Balgobind A.V., Korving J., Proost N., Begthel H. (2019). An organoid platform for ovarian cancer captures intra- and interpatient heterogeneity. Nat. Med..

[48] Vlachogiannis G., Hedayat S., Vatsiou A., Jamin Y., Fernández-Mateos J., Khan K., Lampis A., Eason K., Huntingford I., Burke R. (2018). Patient-derived organoids model treatment response of metastatic gastrointestinal cancers. Science.

[49] Broutier L., Mastrogiovanni G., Verstegen M.M., Francies H.E., Gavarró L.M., Bradshaw C.R., Allen G.E., Arnes-Benito R., Sidorova O., Gaspersz M.P. (2017). Human primary liver cancer-derived organoid cultures for disease modeling and drug screening. Nat. Med..

[50] Yan H.H.N., Siu H.C., Law S., Ho S.L., Yue S.S.K., Tsui W.Y., Chan D., Chan A.S., Ma S., Lam K.O. (2018). A comprehensive human gastric cancer organoid biobank captures tumor subtype heterogeneity and enables therapeutic screening. Cell Stem Cell.

[51] Ganesh K., Wu C., O’Rourke K.P., Adileh M., Szeglin B.C., Wasserman I., Marco M.R., Shady M., Zheng Y., Karthaus W.R. (2019). A rectal cancer model establishes a platform to study individual responses to chemoradiation. bioRxiv.

[52] Lee S.H., Hu W., Matulay J.T., Silva M.V., Owczarek T.B., Kim K., Chua C.W., Barlow L.M.J., Kandoth C., Williams A.B. (2018). Tumor evolution and drug response in patient-derived organoid models of bladder cancer. Cell.

[53] Dekkers J.F., Wiegerinck C.L., de Jonge H.R., Bronsveld I., Janssens H.M., de Winter-de Groot K.M., Brandsma A.M., de Jong N.W.M., Bijvelds M.J.C., Scholte B.J. (2013). A functional CFTR assay using primary cystic fibrosis intestinal organoids. Nat. Med..

[54] Berkers G., van Mourik P., Vonk A.M., Kruisselbrink E., Dekkers J.F., de Winter-de Groot K.M., Arets H.G.M., Marck-van der Wilt R.E.P., Dijkema J.S., Vanderschuren M.M. (2019). Rectal organoids enable personalized treatment of cystic fibrosis. Cell Rep..

