# Supplementary material for: Patient-Derived Head and Neck Cancer Organoids Recapitulate EGFR Expression Levels of Respective Tissues and Are Responsive to EGFR-Targeted Photodynamic Therapy
Source: J Clin Med. 2019 Nov 5;8(11):1880. doi: 10.3390/jcm8111880 (PMC6912517; doi:10.3390/jcm8111880)
Supplement: Supplementary file 1 [file jcm-08-01880-s001.pdf]

Patient-derived head and neck cancer organoids recapitulate EGFR expression levels of respective tissues and are responsive to EGFR-targeted photodynamic therapy

Else Driehuis[1†], Sacha Spelier[1,2†], Irati Beltrán Hernández[3], Remco de Bree[4], Stefan M. Willems[5], Hans Clevers[1,6], Sabrina Oliveira[2,3*]

[1] Oncode Institute, Hubrecht Institute, Royal Netherlands Academy of Arts and Sciences (KNAW) and University Medical Center Utrecht, The Netherlands
[2] Cell Biology, Department of Biology, Faculty of Science, Utrecht University, Utrecht, The Netherlands
[3] Pharmaceutics, Department of Pharmaceutical Sciences, Faculty of Science, Utrecht University, The Netherlands
[4] Department of Head and Neck Surgical Oncology, University Medical Center Utrecht, The Netherlands
[5] Department of Pathology, University Medical Center Utrecht, The Netherlands
[6] Princess Maxima Center, Utrecht, The Netherlands
[†] These authors contributed equally to this work
* Corresponding author. email: s.oliveira@uu.nl tel: +31(0)6 3410 3460

Supplementary Material: Figure S1 and S2.

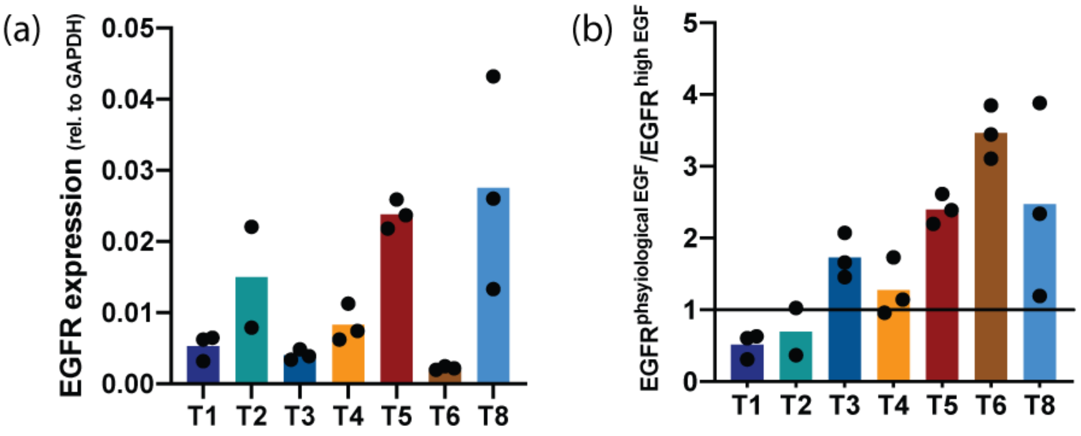

Figure S1. EGFR mRNA expression differs between patient-derived organoid lines and is increased upon medium change to physiological EGF levels. (a) EGFR mRNA expression levels measured in organoid lines derived from different HNSCC patients. Expression is calculated relative to housekeeping gene GAPDH. Experiment was performed in technical triplicate. (b) culturing organoids under physiological EGF resulted in an increase in EGFR mRNA levels in 4/7 lines. EGFR expression is depicted as the ratio of EGFR in medium containing physiological EGF levels, versus EGFR expression in organoid cultured in high EGF medium. When this ratio is > 1, EGFR expression is increased in response to physiological EGF levels.

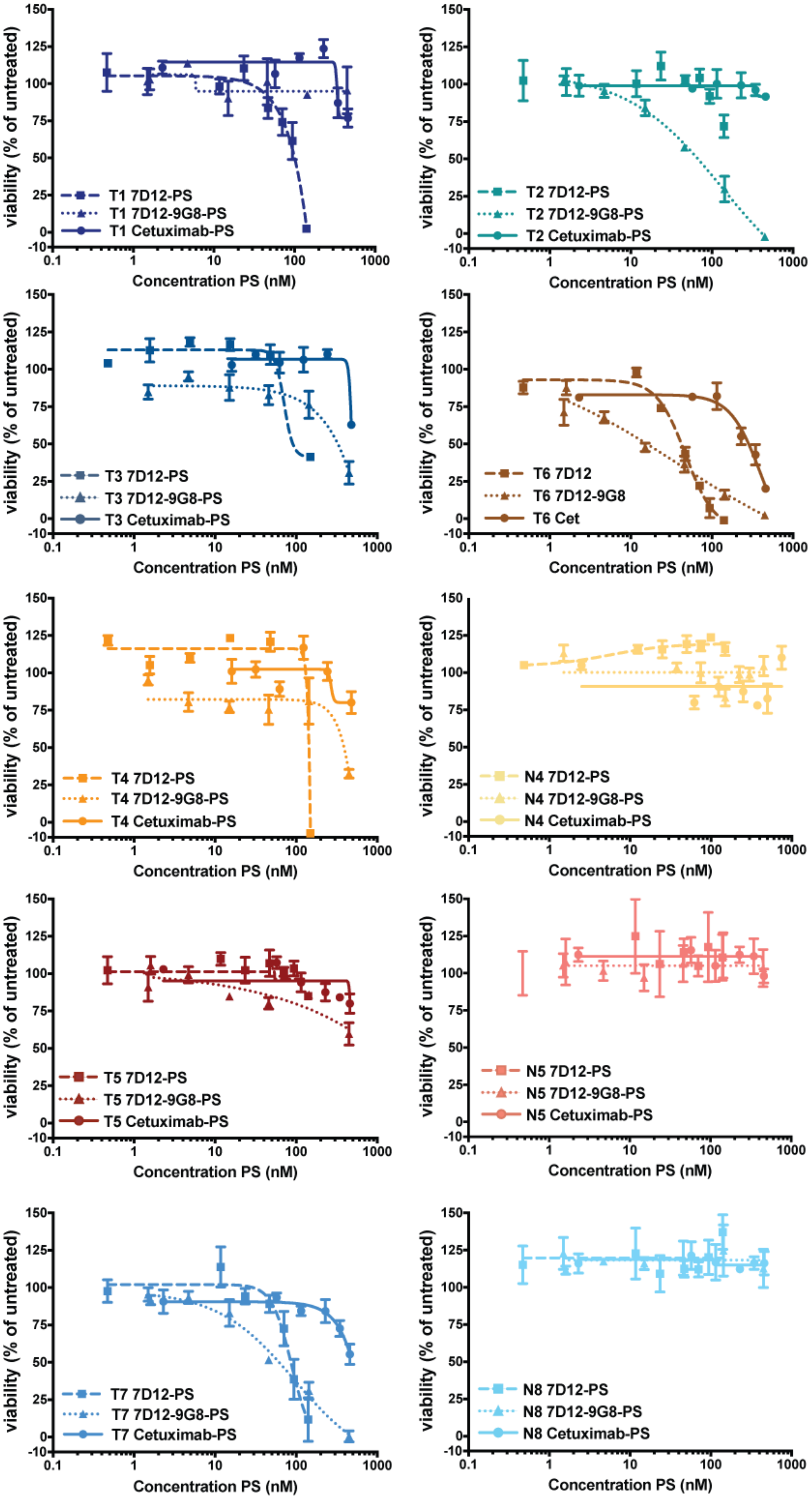

viability (% of untreated)
Concentration PS (nM)
T1 7D12-PS
T1 7D12-9G8-PS
T1 Cetuximab-PS
T2 7D12-PS
T2 7D12-9G8-PS
T2 Cetuximab-PS
T3 7D12-PS
T3 7D12-9G8-PS
T3 Cetuximab-PS
T6 7D12
T6 7D12-9G8
T6 Cet
T4 7D12-PS
T4 7D12-9G8-PS
T4 Cetuximab-PS
N4 7D12-PS
N4 7D12-9G8-PS
N4 Cetuximab-PS
T5 7D12-PS
T5 7D12-9G8-PS
T5 Cetuximab-PS
N5 7D12-PS
N5 7D12-9G8-PS
N5 Cetuximab-PS
T7 7D12-PS
T7 7D12-9G8-PS
T7 Cetuximab-PS
N8 7D12-PS
N8 7D12-9G8-PS
N8 Cetuximab-PS

Figure S2. Comparison of different PS carriers in EGFR-targeted PDT. Results of *in vitro* PDT screens performed with 7D12-PS, 7D12-G98-PS and cetuximab-PS in eight patient-derived HNSCC organoid lines and three matching wildtype organoid lines. Results of 7D12-PS are depicted in dashed lines and squared symbols, results of 7D12-9G8-PS in dotted lines and triangular symbols, and cetuximab-PS in solid lines and circular symbols. Color coding of the different samples is identical to that applied in the main figures.

Supplementary Table S1. details on targeted sequencing of organoids
Used assay: Ion AmpliSeqTM Cancer Hotspot Panel v2+ Next Generation Sequencing
Sequencing performed for research purposes in the UMC Utrecht

*this assay only contains hotspot regions and does not provide information on the full sequence of the depicted genes
**this assay has not officially been validated for detecting amplfications

gene	exons checked by oncopanel
ABL1	4-7
AKT1	3,6
ALK	22-25
APC	14
ARAF	6
ATM	8,9,12,17,26,34,35, 36,39,50,54,55,56,59,61,63
BRAF	11, 15
CALR	9
CDH1	3,8,9
CDKN2A	2
CRAF	6
SDF1R	7,22
CTNNB1	3
EGFR	3,7,15,18-21
ERBB2	19-24
ERBB4	3-5,7-9,15,23
EZH2	16
FBXW7	5,8-11
FGFR1	4,7
FGFR2	5,7,10
FGFR3	7,9,14,16,18
GNA11	5
GNAS	8,9
GNAQ	5
HNF1A	3,4
HRAS	2,3
IDH1	4
IDH2	4
JAK2	14
JAK3	4,13,16
KDR	6,7,11,19,21,26,30
KIT	2,9-11,13-15,17,18
KRAS	2-4
MDM2	6-9
MET	2,11,14,16,19
MLH1	12
MPL	10
MYD88	5
NOTCH1	25,27,37
NPM1	11
NRAS	2-4
PDGFRA	12,14,15,18
PIK3CA	1,4,6,7,9,13, 18,20
PTEN	3,5-8
PTPN11	3,13
RB1	4,6,10,11,14,17
RET	10,11,13,15,16
SMAD4	3-6,8-12
MARCB1	2,4,5
SMO	3,5,6,9,11
SRC	14
STK11	1,4,6,8
TP53	2-10
VHL	1-3
